# Hues of Color Afterimages

**DOI:** 10.1177/2041669520903553

**Published:** 2020-02-18

**Authors:** Jan Koenderink, Andrea van Doorn, Christoph Witzel, Karl Gegenfurtner

**Affiliations:** Justus-Liebig Universität Giessen, Germany; University of Leuven (KU Leuven), Belgium; Utrecht University, the Netherlands; Justus-Liebig Universität Giessen, Germany; Utrecht University, the Netherlands; Justus-Liebig Universität Giessen, Germany

**Keywords:** color, afterimage, complementary colors, supplementary colors, RGB colors, gamma correction

## Abstract

We studied the relationship between color afterimages and complementary colors. The hues of afterimages of 24 inducer hues, uniformly distributed over the rgb color circle, were measured by an iterative method of adjustment. The judgment of equality of hue of the afterimage and a synthesized patch was effectively judged at the moment immediately after the switch-off of the inducer, when the synthesized patch went through any number of iterative adjustments. The two patches—both phenomenally present, but only one optically presented—appeared to the left and right of a fixation mark that was fixated throughout the whole procedure. Thus, both patches were present in eccentric vision. The hues of afterimages were found to be quite different from the hue of the complementary of the inducer. Almost one half of the color circle (orange to chartreuse) leads to afterimage hues in a narrow region of purples. This implies that color circles based on diametrically opposed inducer–afterimage hues are necessarily inconsistent. Yet, perhaps surprisingly, the relation between primary and afterimage hues is still approximately an involution (they are reciprocally related).

## Introduction

If one intently fixates a colored blotch for a while, one becomes aware of a differently colored blotch on extinguishing the inducing (primary) stimulus. This aftermath of the blotch is known as an “afterimage.” It is an often vivid, sometimes vague blotch in visual awareness that persists in the absence of optical stimulation.

In the case of a stabilized retinal image, afterimages cannot even be distinguished from primary images (Burbeck, 1986; Kelly, 1969; Koenderink, 1972). In the unstabilized case (as in this article), the afterimages tend to be somewhat blurred due to micronystagmus during fixation. Even in the unstabilized case, the primary image becomes unsaturated and sometimes is lost (as in stabilized vision) after some period of strict fixation. In any case, after half a minute or so, we observed that the afterimage appears much more vivid than the primary image was immediately before a switch-off. Of course, this initial vivid appearance rapidly deteriorates too, much like that of the primary image. All afterimages reported here are of the “negative” variety.

We consider the hue of the afterimage as a function of the hue of the original “inducer.” This is an issue that has a number of practical applications. For instance, artists routinely use the afterimage (Quiller, 1989) intentionally in order to find the color that is “complementary” (a word with various, mutually distinct meanings) to the inducer. These complementaries also tend to have opposite affective connotations (Albertazzi, Koenderink, & van Doorn, 2015), such as *warm–cool*, so they have important functions in the art of composition. Unfortunately, the literature in both the arts and the sciences is rather ambiguous.

Colorimetrically any color allows of infinitely many “complementaries,” defined as colors that are coplanar (in cie xyz space, say) with the inducer and the (arbitrarily assigned) “achromatic direction,” but of a distinct dominant wavelength (Koenderink, 2010; Wyszecki & Stiles, 1967). It is an awkward concept, as it refers to a fictitious and moreover phenomenological object (the achromatic direction), that ill fits the fully objective nature of colorimetry and varies considerably between observers (Witzel, Valkova, Hansen, & Gegenfurtner, 2011). Thus, this concept is perhaps better avoided.

In the case of object colors, an illuminant is physically and formally implied. Moreover, phenomenologically, a white reference is needed to “anchor” the perceived object colors. In such a context, the notion of a unique “supplementary” object color makes sense (Koenderink, 2010; Ostwald, 1919; Schopenhauer, 1816). It is defined by a spectral reflectance factor that equals one minus the spectral reflectance factor of the original object color. One readily proves that the supplementary is also the complementary with respect to the reference white.

But whereas this notion of “supplementary” is well defined and formally elegant, it is practically useless, because supplementary spectral reflectance factors cannot readily be produced.

Display colors are produced by the incoherent superposition of three spectrally distinct sources, colorimetrically the addition of three color channels (Foley, van Dam, Feiner, & Hughes, 2005; Smith & Lyons, 1996). Hence, display colors also allow of a unique “supplementary.” In this context, the supplementary is simply defined as the color that, when added to the original, yields the display white. This concept of supplementarity is well defined and indeed useful, because the display white is a very relevant key object that tends to be phenomenologically present in many cases. In rich images (say photographs of varied scenes), the “white” is usually implied by the maximum of the red, green, and blue coordinates, although it may also be explicitly present.

For example, “color correction” of images is most readily done by removing color casts in objects that “should” appear white—or gray—in the image. Such anchors are usually identified cognitively; the standard example is the bridal gown in wedding photography, which is hard to miss and in all likelihood represents white (e.g., see https://www.summitprintingpro.com/graphic-design/tutorials/color-cast-removal.html). Rare pink dresses sometimes lead to unfortunate results (faces gain a cyanish cast) when the lab technician is acting in a routine fashion.

A supplementary is obviously also complementary with respect to the chromaticity of the display white. The hue of the afterimage is often considered to be that of the supplementary or the complementary, with respect to a phenomenological “white,” which may be understood as a memory color (Hansen, Olkkonen, Walter, & Gegenfurtner, 2006; Witzel et al., 2011). Perhaps for no good reasons, many people intuitively default to this notion. If one ascribes to that, there are various consequences to accept. For instance, the hue of the afterimage of a color that has the hue of the afterimage of a fiducial color (1 of our 24 inducing stimuli) should be equal to the hue of that fiducial color because the complementary of the complementary is by definition the original. One might call this the issue of reciprocity.

Formally, the map of the space of inducer hues to the space of afterimage hues (in both cases a topological circle) would be an involution, meaning that the map applied twice is the identity—or, equivalently, that the map is its own inverse.

As said, to “find” the complementary hue, artists often use the afterimage. It is often silently suggested—in the arts and sciences—that this method indeed yields that intended result. This is either trivially true (by definition), or it requires an empirical backup.

There are (at least) two issues to be considered:

- is the hue of the afterimage that of a complementary (or supplementary)?
- is the hue of the afterimage of a blotch that has the color of some afterimage the color of the latter’s inducer? That is the issue of “reciprocity.”

Whether reciprocity applies to the afterimage is (or, at least, should be) an empirical issue that—to the best of our knowledge—has only be answered affirmatively in the literature, although usually in a silent mode.

Here, we put it to an empirical test.

## Methods

### Equipment

Stimuli were presented on the lcd screen of an Apple MacBook Pro 15″ (mid-2007 model). The colorimetric parameters of the primary cardinal colors are known from a photospectrometric calibration.

The display was linearized using the Bergdesign Supercal 1.2.4 method and radiometrically calibrated using an X-Rite ColorMunki Photo spectrophotometer. Photometric data of the display are as follows:

**Table:** 

Red	x = 0.5995	y = 0.3406	L = 68.9
Green	x = 0.3259	y = 0.5723	L = 197.4
Blue	x = 0.1534	y = 0.1346	L = 53.2

We do not consider these data are of much relevance, because our results should reproduce on any modern display unit. Because of fundamental colorimetric reasons, all modern display units converge on the same red, green, and blue components, the only difference being in total radiant power and various technicalities.

The screen was viewed from a distance of about 57 cm and subtended about 32° × 20°.

### Participants

A group of 17 participants consisted of PhD students, postdocs, and technical or administrative staff. All volunteered; none had experience with experiments involving color, and various had no experience with formal experiments in vision science. They were tested for normal trichromacy (Ishihara, 1917).

## Experiment

Experiments were performed in a dark room. The task—which is perhaps more involved than in generic psychophysical experiments—was explained, and the initial trials were supervised, after which the participants proceeded on their own.

### Modus Operandi

**Figure 1.**
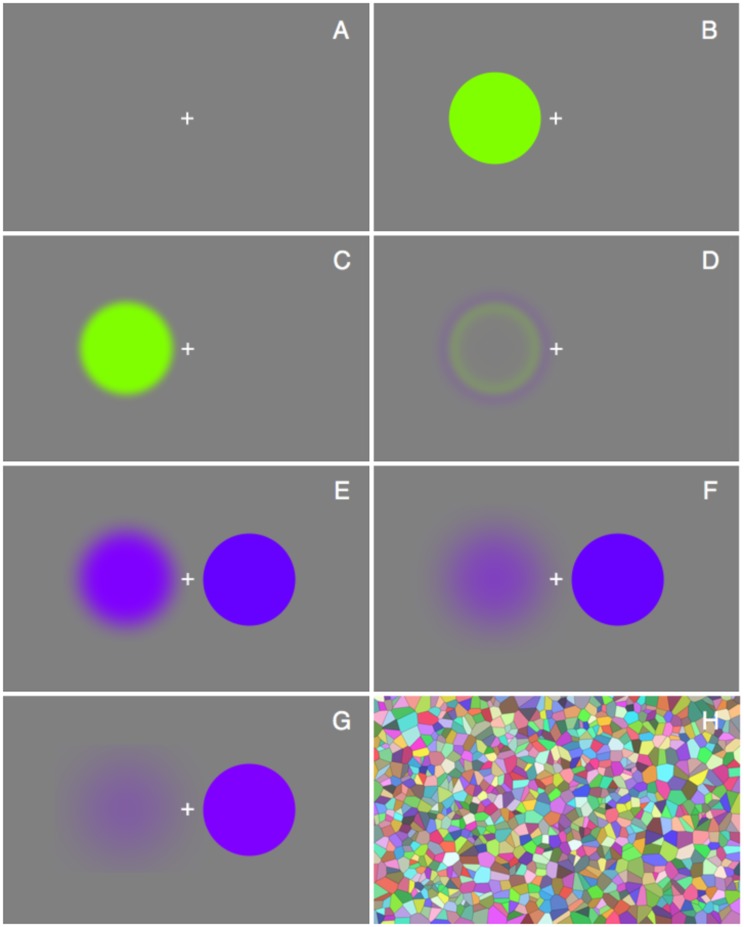
A full cycle of the adjustment procedure. Note that the figure approximates the subjective appearance of the stimuli to the observers. From top to bottom, left to right: (A) Empty screen with fixation mark (mark not on scale); (B) inducer appears as the participant hits the space bar; (C) the inducer grows less vivid; (D) the inducer often gains a complementary rim; for some observers, the inducer is not noticed any more; (E) after at least 20 s, the participant starts the next phase; the inducer vanishes and the afterimage appears; at the same time, the matching patch appears. The participant adjusts the matching patch using the arrow keys; (F and G) when this becomes hard, due to the paling of the afterimage, the participant hits the space bar; (H) the “wipeout” pattern appears for a few seconds, then the empty screen with fixation mark reappears; the participant starts a new round. The only difference is that the setting of the matching patch is retained. Only when satisfied that the match cannot be improved does the participant hit the return key, thus terminating the present trial.

The method is necessarily somewhat involved because the participants have to hunt a fleeting target. While they attempt to match the hue of the afterimage, that image is always in the course of fading. After even a short time, a comparison becomes doubtful. This means that for any match the afterimage needs to be refreshed several times. Each time, the match is improved a bit and so the participant iterates step by step toward a satisfactory match. The general layout and the time course of a typical trial are schematically suggested in Figure 1.

Participants fixate the center of the screen (a fixation mark being provided). The background was gray at half the luminance of the display white, thus *x* = 0.3123, *y* = 0.3390, *Y* = 159.8. The inducer and synthesizer patches have a diameter of about 8°, their centers being at about 5.3° from the central fixation mark.

The participant starts a trial by hitting the space bar, at which a randomly chosen inducer appears on the left. Instructions are to fixate the center continuously. After 20 s, a beep reminds the participants that an action is expected. The response mode is triggered by the participant by hitting the space bar; this extinguishes the inducer and displays another blotch at the right. The hue of that latter patch can be adjusted using the arrow keys of the keyboard (Koenderink, van Doorn, & Ekroll, 2016). A key driven interface is necessary because of the fleeting nature of the visual impressions. This allows the participants to fixate the central mark at all times, whereas a mouse-controlled graphical interface would prevent that.

In this experiment, the participants can only control the hue of the right-hand blob with the left–right arrow keys, whereas the intensity and saturation are fixed. We initially attempted to make more complete matches, but this proved to be beyond the capabilities of our participants. However, no participant complained that the matching of hue was intrinsically difficult or ambiguous. The main problem was the limited time available due to the time course of the afterimage (see Appendix A).

Maintaining fixation, the participant adjusts the hue of the blotch at right using the left–right arrow keys. The task is to match the hue of the afterimage, which is present in visual awareness at the left, even though the physical stimulus has been extinguished. A conceptual complication is that the participant adapts to the matching stimulus too, of course.

When the afterimage dwindles, the space bar can be used to switch back to the inducer. First, a “wipeout” screen appears for 2 s, and after another 20 s of adaptation, the adjustment may proceed. The wipeout screen is illustrated in Figure 1. It consists of a large number of randomly colored patches, whose mean chromaticity and luminance equal that of the gray adapting background. Its texture elements were much finer than the size of the stimulus patches, and it did not elicit any patterned afterimages. It was on for 2 s. The matching stimulus then appears in the same hue as it had in the immediately preceding iteration. This allows a progressive approach of the target through the course of any number of iterations.

The only “clean” comparison is at the moment immediately after the switch, when the afterimage is most vivid and the matching stimulus has not yet started to adapt. It is only a fleeting moment that was prepared for by the (sometimes many) earlier iteration cycles.

When the participant judges that the hue of the right blotch matches that of the afterimage, the participant may mark that decision by hitting the enter/return key. This terminates the iteration cycle.

At that event, the wipeout screen appears, followed by a new (randomly chosen) inducer for the next trial. After 24 of such trials, the task is done, and the program quits.

We used 24 inducers spaced uniformly on the display color circle, based on the yellow–green–cyan–blue–magenta–red periodic sequence (see Appendix A). Intermediate steps are obtained by linear interpolation. (For instance, the yellow–green range is covered by the rgb display coordinates {1−ξ,1,0}, where the parameter *ξ* runs from zero to one.) This yields the most vivid colors that can be obtained in an rgb–display context, and thus also the most vivid afterimages. Full colorimetric specifications of all stimuli are given in Appendix C. The stimuli were not equated with respect to luminance. We think, maybe counter to conventional wisdom, that equating luminance is not the proper approach for studying color. After all, luminance basically excludes any contribution by the S-cones, and a world without S-cone activation would indeed be much less colorful. However, our stimuli were approximately equated in terms of perceived brightness (see Koenderink, van Doorn, & Gegenfurtner, 2018).

The starting color was randomly picked from a wide region about the complementary. For instance, for yellow, it was in the range cyan to magenta, for green, it was in the range blue to red, and similarly for the other inducers.

All participants completed a session in about an hour.

### Observations

**Figure 2.**
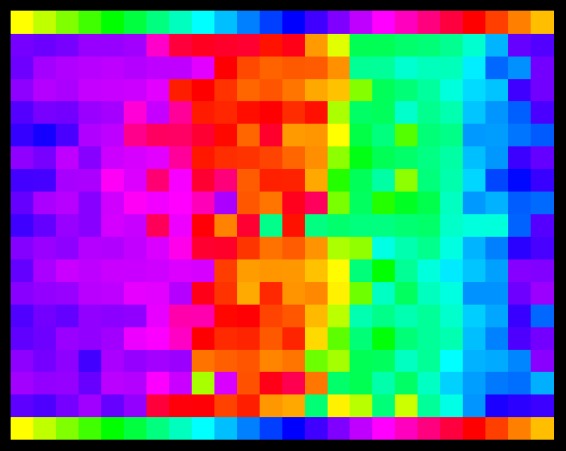
An overview of all responses. The rows at top and bottom show the inducer hues. This matrix plot shows the hues of the afterimages indicated by the participants as a function of the inducer hue.

An overview of all settings for all participants is provided in Figure 2. This is perhaps a slightly complicated figure, so we discuss it in some detail.

The matrix plot shows the results of all trials (all inducers, all participants), a total of 17 × 24 = 408 trials. To get ones’s bearings, notice that the rows at top and bottom show the inducer hues.

In order to obtain an overall result, we use robust circular statistics (Appendix B). For each inducer, we obtain a cloud of 17 points on the hue circle, of which we determine the median and the interquartile range. This effectively deals with the outliers that are evident in Figure 2.

**Figure 3.**
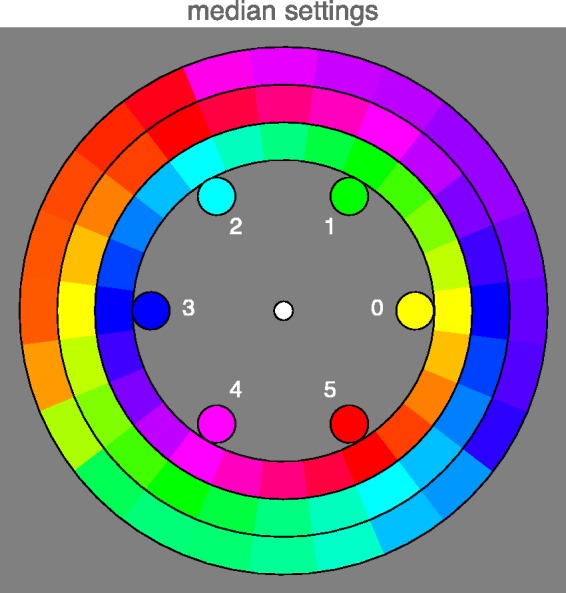
The median response for all observers (outer annulus). The inducers (inner annulus) and supplementary hues (center annulus) are shown for comparison. Color indices for the cardinal colors (notion of “cardinal colors” explained in Appendix A) are indicated, although all 24 (mostly interpolated) hues are shown. Notice the apparent clustering of the afterimage hues.

This result can be represented in various ways, perhaps the most obvious one is displayed in Figure 3. Here, the inducers are shown in the inner annulus, the hues of the supplementaries in the middle annulus, and the median afterimage hues in the outer annulus.

The median interquartile range is 1.1 (25% quartile 0.83, 75% quartile 1.51), a little more than a single step on the 24-step scale. This indicates that the choice of inducers (a 24-step hue circle) was appropriate. The observer spread is somewhat less than that reported by Bagley and Maxfield (1986).

**Figure 4.**
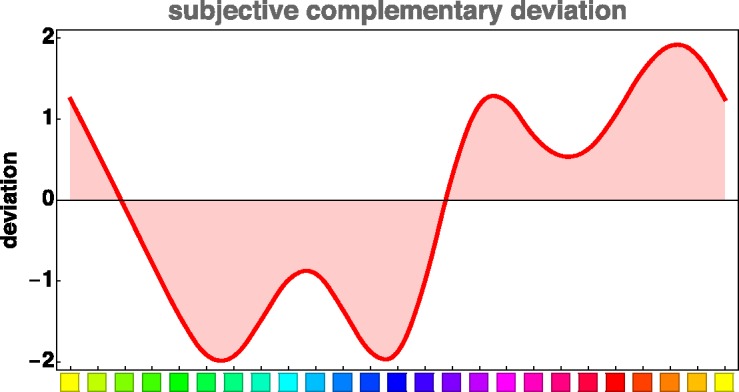
The medians of the difference between the hue indices (see Appendix A) of the afterimages and the corresponding supplementaries of the inducers, as a function of the inducers, for all participants. For this graph, the data were slightly smoothed; for the case of all other figures, no such smoothing was applied.

Another, perhaps more precise, though less intuitively informative, way to visualize the same results is Figure 4. Here, we show medians of the difference between the hue indices of the afterimages and the corresponding supplementaries of the inducers for all participants. The data were lightly smoothed using a Gaussian kernel with a spread parameter of one step on the 24-step scale. (The smoothing has little effect on overall shape but effectively removes minor high frequency variations that are obviously “noise.”)

**Figure 5.**
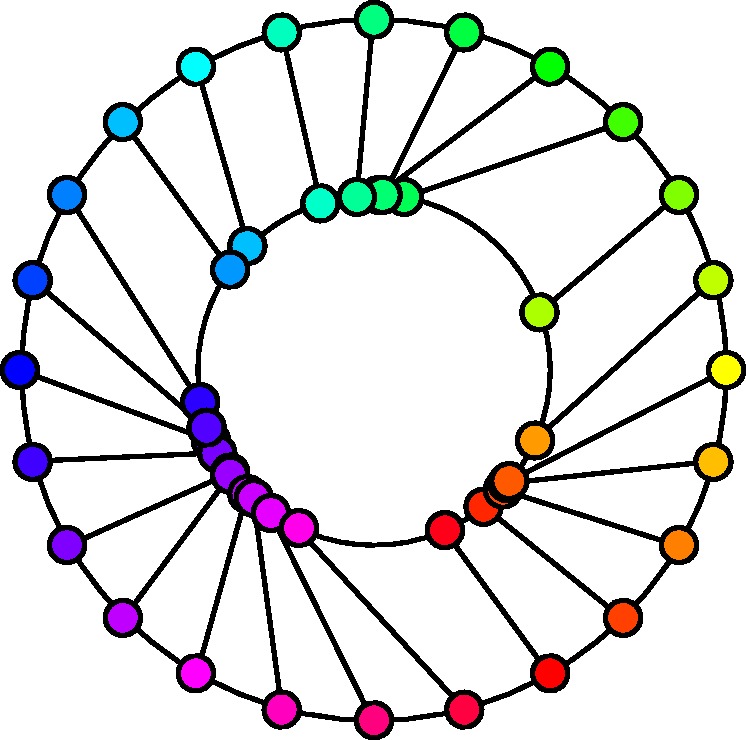
A shift diagram for the pooled data of all participants. The outer circle represents the hues of the supplementaries of the inducing colors, whereas the inner circle shows the corresponding hues of the afterimage. For ease of reference, corresponding points have been joined by connecting line segments.

Yet another representation of the same data (see Figure 5) lets one visualize important structure in the data that is not immediately obvious from the previous representations.

This shift diagram very clearly shows the relation between the hues of the supplementary colors of the inducers and the hues of the afterimage colors.

## Analysis

According to Figure 4, there exist only two inducers whose afterimages correspond to their supplementary colors (zero deviations in Figure 4). One is the inducer with a color index (Appendix A) of 0.5, which is a little greener than “unique yellow,” but certainly yellower than “unique green,” and which we call “chartreuse.” The other inducer has a color index of 3.5 and corresponds to a bluish purple. For all other hues, the afterimage hues fail to be supplementary.

**Figure 6.**
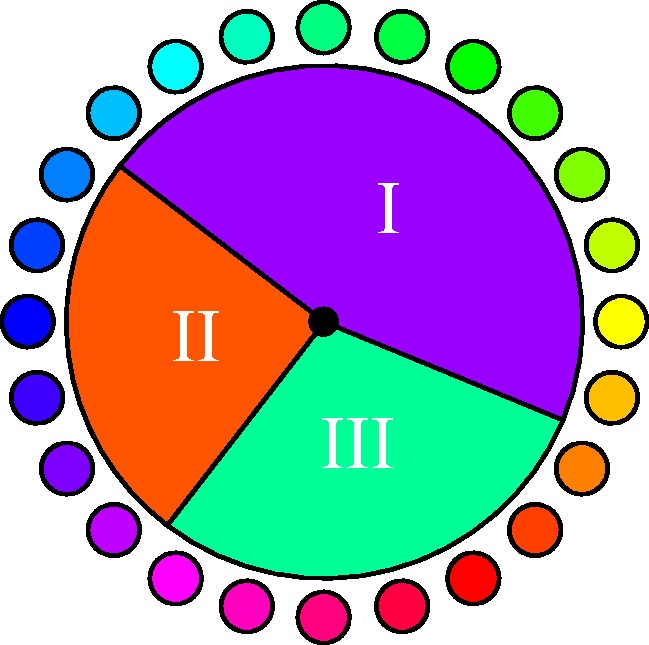
The division of the hue circle induced by the three clusters. The steps along the color circle indicate the inducers. The colors of the sectors are the centers of the afterimage clusters.

From the shift diagram (Figure 5), one gleans that there is an apparent clustering of the afterimage hues. This is indeed immediately borne out on running a clustering algorithm. We used the angular difference as the distance function. Many clustering methods yield essentially identical results (here we show results for the method of “k-means”); the clustering is very robust. As summarily shown in Figure 6, there are three clusters of mutually connected parts of the color circle. Notice the fact that the clusters are connected index ranges is not trivial, as the cluster analysis treats the indices as mere labels, without any topological structure. Thus, this suggests that the clusters are likely to be meaningful.

Cluster 1 covers the color index range of the inducers from –0.3 to 2.4, Cluster 2 the range from 2.4 to 3.9, and Cluster 3 the range from 3.9 to 5.7 (= –0.3).

**Figure 7.**
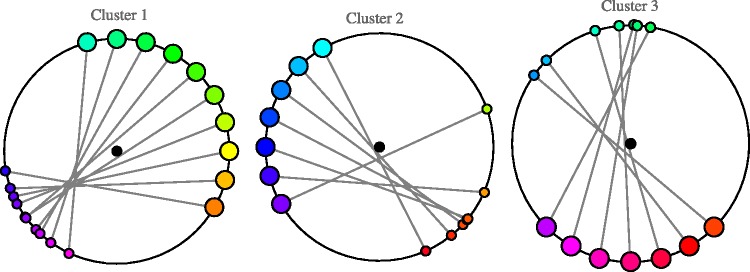
The clusters for all participants. The large circles indicate the inducers. The small circles indicate the afterimages. This figure (together with Figure 6) serves as a convenient summary of the results.

The structure is perhaps better explained in Figure 7. The structure for Cluster 1 is clearest; here, a large range of inducers yields very similar hues in the afterimage.

A similar contraction is seen to happen to the other clusters, although perhaps less pronounced. A very coarse-grained summary might mention just three afterimage hues, due to three ranges of inducer hues. (This appears to fit suggestions by Pridmore, 2007.) Although indeed overly schematic, it approximately captures the essence of the phenomenology quite well, certainly better than the common notion that afterimage hues are given by the supplementary hues.

**Figure 8.**
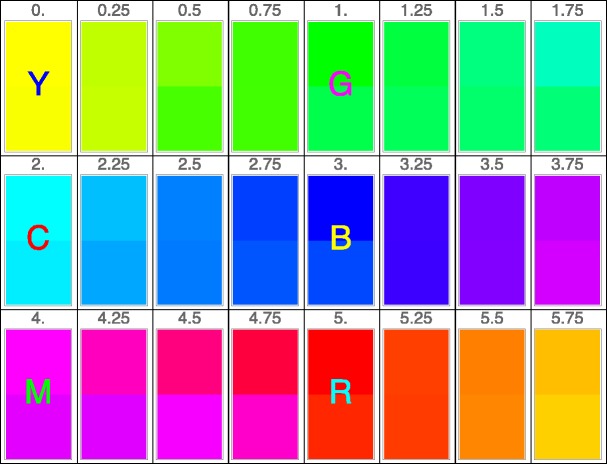
Here, we show the fiducial colors of the 24-step scale on top and the hue of the afterimage of the color that has the hue of the afterimage of the fiducial color at the bottom. It shows that the relation is fairly close to an involution, although admittedly only approximately.

The two inducers with supplementary afterimages, chartreuse (Cluster 1) and blue-purple (Cluster 2), are also approximately reciprocal, that is, chartreuse produces an afterimage close to blue-purple, and vice versa. It is difficult to inspect reciprocity for other inducers due to their deviations from supplementary colors. According to reciprocity, the inducer (A) that has the color of an afterimage of another inducer (B) should elicit an afterimage that corresponds to that other inducer (B). Let’s call that “other inducer” (B) the “counter-inducer.” We identified the afterimages of counter-inducers (B) using the deviations from supplementaries shown in Figure 4. From the relation shown in Figure 4, we obtain a smooth (interpolation) function Φ:S↦S of the hue circle to itself such that $h_{a}=\Phi (h_{i})$ for the hue of the inducer *h_i_* and the hue of the afterimage *h_a_*. Reciprocity would imply $h_{a}=\Phi (h_{i})\to h_{i}=\Phi (h_{a})$, thus $\Phi^{-1}=\Phi$, that is to say, the function would be its own inverse (an “involution”). Equivalently, iterating the relation once Φ o Φ should result in the identity, Φ o Φ=I. Consequently, $\bar{h}=\Phi \left(\Phi (h_{i})\right)$ should yield $\bar{h}=h_{i}$ within the empirical uncertainty (that is $\sqrt{2}$ times the standard deviation). A more intuitive way to visualize possible reciprocity failure is to compare the hues of a number of such $\bar{h}=h_{i}$ pairs distributed over the hue circle. In Figure 8, a number of equally spaced pairs have been conveniently juxtaposed. Apparently, reciprocity holds quite well on the whole, although some deviations can certainly be made out. The yellow–blue diameter of the hue circle maps on itself under the iterated function Φ o Φ, but deviations are especially visible in the purple range and in the cyan range. However, in view of the empirical spread, we cannot reject reciprocity.

## Discussion

In sum, our results indicate that most afterimages do not correspond to the supplementaries of the inducers. What is perhaps most striking in the present findings is that there is only one unique pair that corresponds to supplementary colors and is at the same time reciprocal. This is the chartreuse (0.5)/blue-purple (3.5) pair (Figure 4). In addition, afterimages were condensed in three regions of color space so that they correspond to three clusters (Figure 7). When comparing inducers to the afterimage of “counter-inducers,” reciprocity approximately holds for most colors (Figure 8). However, deviations from reciprocity might occur near yellow, turquoise, and reddish purple.

One phenomenon that could have contributed to deviations of afterimages from supplementary colors is the Abney effect (see Wyszecki & Stiles, 1967). Our inducer stimuli and the matching stimuli had different degrees of saturation. Changes in saturation can lead to differences in perceived hue. Could this be the sole reason for our results? We do not think that this is the case. First, the change in perceived hue with increased saturation that is termed Abney effect mostly occurs at very high levels of purity, outside the gamut of display monitors. Second, the biggest Abney effects are in the long-wavelength end of the spectrum (reddish lights), which is not where we see the biggest deviations from supplementarity (Figure 8).

Our observations clash with much of the earlier (or even” historical”) literature on complementary colors, although that literature does not always make precise statements about the hues of afterimages. For example, Goethe’s concept of complementary colors or Hering’s opponent colors describe complementary colors through the opposition of red and green, and blue and yellow. If these approaches predicted afterimages, red–green and yellow–blue should be reciprocal inducer pairs (Hering, 1905–1911; von Goethe, 1993). However, this is not the case. Instead, we observed that green induced purple, red induced blue, blue induced red-orange, and yellow induced blue-purple (Figure 7).

**Figure 9.**
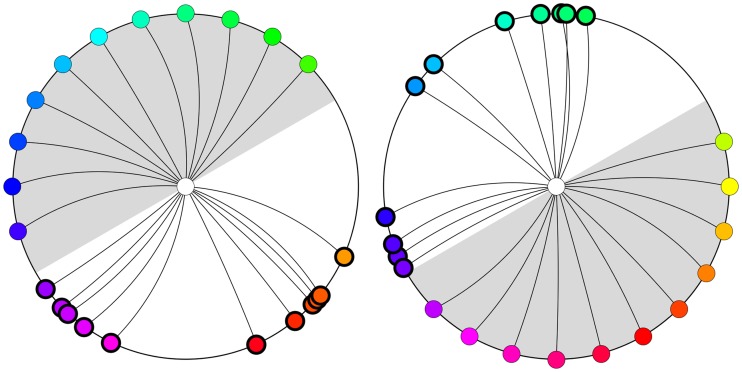
Here, one half of all inducers are connected to the opposite half of the hue circle by circular arcs through the center to the median afterimage hues. This is a plot similar to Figure 9 in Wilson and Brocklebank. Different from the latter, we find no perfect “afterimage pairs” (the reciprocity issue). Thus, we split the graph in two halves to improve its legibility. Because arcs are forced to pass through the center, this graph illustrates the deviations from the supplementary hue (the antipode) especially well.

The claim that afterimages correspond to supplementary colors may still be found in contemporary references (Manzotti, 2017). However, several other sources seem to have made observations similar to ours, although they—unfortunately—rarely said or acknowledged them in writing: Aubert (1865) seems to have been the first to notice that the hues of afterimages are often different from the supplementary, a notion that was taken as almost self-evident by von Helmholtz (1867). Like ours, the study by Wilson and Brocklebank was focused on hue matching, although they also varied the saturation. They used colored papers and rotating disks (Wilson & Brocklebank, 1955), a verily Herculean task. These authors claim that reciprocity holds within the experimental uncertainty, but they clearly show the deviations of afterimage hues from the supplementary hues. It seems that the results of these authors are at least qualitatively close to the present result. Our Figure 4 is comparable to their Figure 5, our Figure 9 to their Figure 9. Phuangsuwan, Ikeda, and Mepean (2018) recently reported similar results along these lines. That opponent color theory fails to account for the present findings was detailed by Pridmore (2007).

Our observation that afterimages are approximately reciprocal (Figure 8) is in line with the above literature. However, the observation of the pronounced clustering of the afterimage hues for about half of the color circle suggests that reciprocity is largely irrelevant. This is best appreciated from Figure 7 (left). Almost half of the color circle (orange to cyan) has a very similar hue of afterimage (a purple) and—conversely—the afterimages of purples are ill-defined; they may be either oranges or chartreuses. These observations change the potential relevance of “reciprocity” in a perhaps unexpected way: They destroy the formal importance of the afterimages as indicator of antipodality of the hue circle and hence render the notion of reciprocity perhaps of less interest as a generic property.

If we are right on these conclusions, then *any* color circle-like representation that purports to represent afterimage hue pairs as diametrically opposites (Bagley & Maxfield, 1986) has to be inconsistent. This is true for both the rgb supplementarity (suggesting the basic pairs R–C, G–M, and B–Y) and for the Hering opponent pairs (suggesting the basic pairs R–G and Y–B). With some adjustments—using curved arcs instead of straight diameters (Figure 9)—the supplementary color scheme can still be accommodated, but the Hering color scheme fails anyway (Pridmore, 2007; Wilson & Brocklebank, 1955).

However, the idea that supplementary colors may be arranged along curved lines is of little use for the application in the arts. In the arts, it makes obvious sense to have colors and their supplementaries diametrically opposite to each other, as they will mutually add to white. This is the basis of most hue circle constructions in the arts, like the Quiller (1989) wheel, which is in widespread use. Artists using the electronic medium (currently typical for applied art as in illustration and commercials, as well as computer [implying “computed”] graphics) will generally use a gamma of (roughly) 2.2. One expects this to have no effect on the afterimage hues. We actually checked this with a group of 20 participants (volunteers from Giessen University, thus all different from the group discussed in this article) and found that this expectation bears out; the results are not significantly different from those reported here. Taken together, since afterimage hues and supplementary hues are generically mutually distinct, the afterimage hue cannot be represented in the conventional artist’s hue circle.

Our experiment focused on the *hue* of afterimages. Additional aspects of afterimages, like vividness, may also vary across hues (Brücke, 1851; Burckhardt, 1866; Fechner, 1838, 1840; von Goethe, 1993; von Helmholtz, 1867; Zeki, Cheadle, Pepper, & Mylonas, 2017). Although many afterimages of vivid colors are also very vivid, *some are not*. In our experiment, participants often remarked on the variation of vividness because it made their task difficult. The variation of vividness may well depend upon the display (Manzotti, 2017); for example, in our case, the green channel is rather desaturated. More importantly, the vividness of the afterimage depends also on the gray level of the uniform background. For example, it makes a difference whether we use a white instead of a midgray (as used here) background. Thus, the gray level should really be included as a parameter, which would imply a major undertaking. The vividness of afterimages is evidently an issue that requires further investigation. Indeed, it cannot be said that the mechanism of negative afterimages is fully understood (Anstis, 2017).

## Figures and Tables

**Figure A1.**
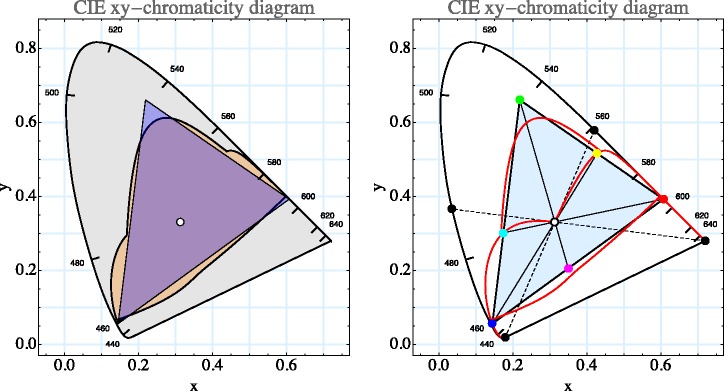
The RGB primaries (“parts of white”) in the chromaticity diagram. At left, we show the RGB triangle based on the “parts of white” (bluish) and the locus of most vivid hues (outline of the brownish region). At right, we indicate the skeleton structure. The red curves that connect the spectrum limits to the white point are the Goethe *Kantenfarben* (edge colors). These provide parts of the locus of most vivid hues. The remaining parts are band-pass Schrödinger optimal colors (green-branch) and their supplementaries, the band gap Schrödinger optimal colors (purple-branch). The spectrum limits and the complementary wavelengths of the spectrum limits are indicated in black. The latter nearly indicate the cut loci for the “parts of white.” Notice that the chromaticity diagram is a central projection from the black point, which severely distorts the geometry and fails to be intuitive to many people.

**Figure A2.**
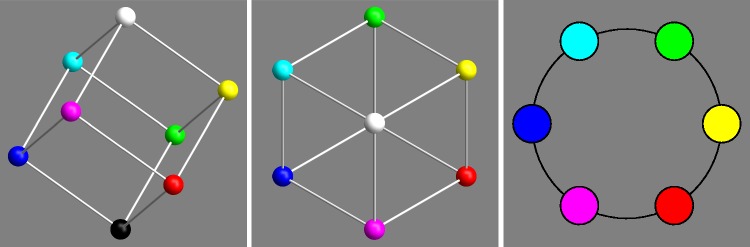
Left: A generic view of the RGB cube. The cube is a unique colorimetric object that depends on the choice of illuminant spectrum. The cardinal colors are located on a nonplanar polygon that surrounds the gray-axis. Center: A view of the RGB cube along the gray-axis, looking toward white. In this orthographic projection, the cardinal colors are arranged as the vertices of a regular hexagon. All full colors (defined by the maximum distance from the gray-axis) lie on the edges of the polygon and can be obtained by linear interpolation between two adjacent cardinal colors. Right: In various conventional representations, the hexagon is rendered as the “hue circle.” This is indeed a pure convention, unlike the colorimetric structure shown at center and left.

## Appendix A: The RGB Colors

In this article, we concentrate on object colors throughout. This implies a standard illuminant and a white reflectance standard. Given the standard illuminant, the Schrödinger object color solid is colorimetrically uniquely determined (Bouma, 1948; Centore, 2017; Koenderink, 2010; Schrödinger, 1920). The white standard is required because object colors require a white “anchoring.” In practice, one may use cie illuminant d65, “average daylight,” whereas the white standard might be a piece of chalk or blotting paper (more officially a pressed ${\text{BaSO}}_{4}$ powder surface). The reasons are not just colorimetric but involve ecological physics, physiology, and ethology. Colorimetry proper has no principled way to define an “achromatic” beam; the object color white comes from ecological physics and enables the constructions discussed here.

Starting from the Schrödinger object color solid, one defines a unique colorimetric basis. The primaries are “parts of white” (Koenderink, 2010; Schopenhauer, 1816), defined by the spectral cut loci 483 nm and 565 nm. In Figure A1, we show these primaries in the cie xy chromaticity diagram (Wyszecki & Stiles, 1967).

The black points in the diagram are the spectrum limits and their complementaries. The complementaries, 487 nm and 561 nm, are very close to the cut loci of the parts of white. The parts of white are shown as a red dot (ir spectrum limit to 565 nm), a green dot (565 nm to 483 nm), and a blue dot (483 nm to the uv spectrum limit).

The boundary of the color solid maps on the full interior of the chromaticity diagram (Figure A1, left). The boundary of the brownish-tinted region contains the Schrödinger full colors. The Schrödinger full colors lie on the boundary of the color solid and have a maximum distance to the achromatic axis; thus, technically, they are the most colorful colors in the sense that all other colors (even on the boundary of the color solid) have some white and/or black content. It is the colorimetric equivalent of the “color circle.” The region between the spectrum locus and the brownish-tinted region contains shades, whereas the brownish-tinted region contains tints. (It is perhaps not superfluous to remark that the spectrum locus represents *black* object colors, a fact that is commonly misrepresented, even in textbooks.)

The red curves shown in Figure A1 (right) show the structure in more detail. The curves that run from the spectrum limits to the white point represent the low-pass and high-pass spectra, the Goethe *Kantenfarben* (“edge colors”).

The rgb triangle subtended by the red, green, and blue parts of white is the optimum for display units in the sense that it claims the largest volume of the color solid. (Notice that volume ratios are colorimetric invariants; no metric is needed.)

The triangle is a very good approximation to the locus of Schrödinger full colors; it is the optimum gamut for rgb representations. Indeed, virtually all modern display units converge on this choice.

The lines through the white point meet the triangle in the secondary cardinal colors, cyan, magenta, and yellow. The six cardinal colors together define a unique (fully determined once the illuminant has been decided on) six-step color circle (Figure A2). The term *cardinal colors* derives from the fact that it is the unique minimal set of colors that generates the full display gamut as its convex hull.

It is convenient to consider the cardinal colors as anchor points in interpolated scales like the 24-step scale used in this study. Thus we define:

**0 (Y)** yellow

**1 (G)** green

**2 (C)** cyan

**3 (B)** blue

**4 (M)** magenta

**5 (R)** red

These color indices are to be understood *modulo 6*, reflecting the closing of the hue circle. Thus, red has not only the color index 5, but just as well –1 (5 – 6) or 11 (5 + 6) and so forth. For interpolated steps, we use decimal fractions. Thus, color index –0.5 (between red and yellow) indicates orange.

This is the unique, natural representation of object colors. It has been reinvented various times (Bouma, 1948; Ostwald, 1919) in colorimetry. It was reinvented by Ray Alvey Smith (Smith & Lyons, 1996) as the HUE-WHITENESS-BLACKNESS (HWB) model in the context of computer graphics. Earlier, Smith had also introduced various color models (like HUE-SATURATION-VALUE [HSV]), but in the 1996 paper, he calls these “flawed.” The hwb is a digital implementation of the Ostwald system, although Smith did not notice that. In this study, we use the hwb system, but the colorimetric derivation summarized in this appendix indicates that it is by no means an ad hoc choice.

Notice that the cardinal colors red, green, blue (“primary cardinal colors”) and cyan, magenta, and yellow (“secondary cardinal colors”) appear as the vertices of a regular hexagon. That this is very different from the rgb *triangle* (Figure A1) with the primary colors as vertices and the secondary colors on the edges is due to the (to many confusing) nature of the chromaticity diagram. Indeed, a projection from the white point would show the secondary colors as vertices and the primary colors on the edges. In order to obtain a consistent geometrical picture, Figure A2 is perhaps to be preferred over the conventional cie–xy chromaticity diagram. It even has a metric if one understands the rgb crate as the unit cube, whereas chromaticity diagrams are projective planes that do not admit of either distance or angle metrics. (This metric represents really Ostwald’s “Principle of Internal Symmetry”; Bouma, 1948; Koenderink, 2010.)

More articulated color circles are made by linear interpolation between adjacent primary and secondary cardinal color pairs. That is how the 24-step color circle used in this study is constructed (further explained in Figure A2).

## Appendix B: Robust Circular Statistics

The responses are represented as a set of unit vectors $r_{i}$, i=1⋯N, representing a cloud of *N* points on the hue circle. The number *N* is the number of observers, the vectors $r_{i}$ the individual responses, which are the settings of the afterimage hue. The mean of the vectors is a point **m** inside the unit circle. The circular standard deviation is conventionally defined as $\sigma =\sqrt{-2\log(1-\text{m})}$, where m=||m|| (Fisher, 1993).

Because this spread *σ* tends to be much smaller than one in all cases, it makes sense to represent the points by their angular separation from the mean.

$$\varphi_{i}=\arctan\left(\frac{(\text{J}\frac{m}{m})\cdot r_{i}}{\frac{m}{m}\cdot r_{i}}\right)$$

where $\text{J}=\left(\begin{array}{l} 0-1\\ 1\;0 \end{array}\right)$ represents a $\frac{\pi }{2}$ counterclockwise turn.

Notice that this works out in a consistent fashion because the method avoids circular wraparound problems, which is again possible because σ≪1.

The quartiles of $\varphi_{i},\;i=1\cdots N$ then define the median relative to $\frac{m}{m}$ and the interquartile range. All data are presented in this way.

## Appendix C: Stimulus and Matches Color Specifications

**Table C1.** F for “fiducial,” R for “response.” “hi” hue-index (0…24); “{R,G,B}” display red–green–blue (on [0, 1]); “{x, y, L}” the CIE xyL coordinates.

F	R	F			R			F			R		
hi	hi	R	G	B	R	G	B	x	y	L	x	y	L
0	13.59	1	1	0	0.40	0	1	0.4270	0.4866	266.3	0.2287	0.1694	80.5
1	13.87	0.75	1	0	0.47	0	1	0.4095	0.5015	249.1	0.2395	0.1744	85.4
2	14.39	0.50	1	0	0.60	0	1	0.3879	0.5198	231.9	0.2578	0.1828	94.3
3	14.42	0.25	1	0	0.60	0	1	0.3609	0.5427	214.6	0.2588	0.1833	94.8
4	14.96	0	1	0	0.74	0	1	0.3259	0.5723	197.4	0.2759	0.1912	104.1
5	15.15	0	1	0.25	0.79	0	1.01	0.2875	0.4748	210.7	0.2817	0.1938	108.2
6	15.61	0	1	0.50	0.92	0	1.02	0.2631	0.4129	224.0	0.2946	0.1998	117.4
7	16.32	0	1	0.75	1.02	0	0.94	0.2462	0.3700	237.3	0.3127	0.2082	119.7
8	19.59	0	1	1	1	–0.02	0.09	0.2338	0.3386	250.6	0.5408	0.3049	70.3
9	20.60	0	0.75	1	1	0.14	–0.01	0.2216	0.3078	201.3	0.5569	0.3899	95.0
10	21.14	0	0.50	1	1	0.29	0	0.2058	0.2676	151.9	0.5100	0.4164	125.2
11	21.33	0	0.25	1	1	0.33	0	0.1843	0.2130	102.6	0.5003	0.4246	134.7
12	21.40	0	0	1	1	0.35	0	0.1534	0.1346	53.2	0.4972	0.4273	138.1
13	22.43	0.25	0	1	1	0.61	0	0.2040	0.1580	70.4	0.4602	0.4586	189.0
14	1.29	0.50	0	1	0.68	1	0	0.2443	0.1766	87.7	0.4038	0.5064	244.1
15	5.33	0.75	0	1	0	1	0.33	0.2771	0.1917	104.9	0.2782	0.4513	215.1
16	5.89	1	0	1	0	1	0.47	0.3044	0.2043	122.1	0.2653	0.4184	222.6
17	5.77	1	0	0.75	0	1	0.44	0.3343	0.2182	108.8	0.2679	0.4250	220.9
18	6.35	1	0	0.50	0	1	0.59	0.3791	0.2388	95.5	0.2565	0.3961	228.7
19	7.16	1	0	0.25	0	1.01	0.80	0.4531	0.2730	82.2	0.2439	0.3643	241.0
20	9.00	1	0	0	0	0.75	1	0.5995	0.3406	68.9	0.2216	0.3077	201.1
21	9.64	1	0.25	0	0	0.59	1	0.5177	0.4098	118.3	0.2121	0.2836	169.9
22	12.68	1	0.50	0	0.16	–0.01	1	0.4736	0.4472	167.6	0.1857	0.1460	61.8
23	13.29	1	0.75	0	0.32	0	1	0.4460	0.4706	217.0	0.2166	0.1638	75.4
